# Stroke Management in the Intensive Care Unit: Ischemic and Hemorrhagic Stroke Care

**DOI:** 10.3390/neurosci6040121

**Published:** 2025-11-26

**Authors:** Aleksandar Sič, Vasilis-Spyridon Tseriotis, Božidar Belanović, Marko Nemet, Marko Baralić

**Affiliations:** 1 Faculty of Medicine, University of Belgrade, 11000 Belgrade, Serbia; 2 Department of Neurology, Agios Pavlos General Hospital of Thessaloniki, Leoforos Ethnikis Antistaseos 161, Kalamaria, 55134 Thessaloniki, Greece; vasilistseriotis@hotmail.com; 3 Laboratory of Clinical Pharmacology, Aristotle University Campus, Aristotle University of Thessaloniki, 54124 Thessaloniki, Greece; 4 Department of Neurology, Helios Amper-Klinikum Dachau, Krankenhausstraße 15, 85221 Dachau, Germany; bozidar.belanovic@gmail.com; 5 Department of Anesthesiology and Perioperative Medicine, Mayo Clinic, 200 1st St SW, Rochester, MN 55905, USA; nemetmmm@gmail.com; 6 Clinic of Nephrology, University Clinical Center of Serbia, Pasterova 2, 11000 Belgrade, Serbia

**Keywords:** stroke, stroke management, acute care, prophylactic care, intensive care unit

## Abstract

Stroke is the second-largest cause of death and disability worldwide, and many patients require intensive care for airway compromise, hemodynamic instability, cerebral edema, or systemic complications. This review summarizes key aspects of ICU management in both acute ischemic stroke (AIS) and hemorrhagic stroke (HS). Priorities are airway protection, oxygenation, individualized blood pressure targets, and strict control of temperature and glucose. Neurological monitoring and prompt management of intracranial pressure (ICP), together with timely surgical interventions (hemicraniectomy or hematoma evacuation), are central to acute care. Seizures are treated promptly, while routine prophylaxis is not recommended. Prevention of aspiration pneumonia, venous thromboembolism, infections, and other intensive care unit (ICU) complications is essential, along with early nutrition, mobilization, and rehabilitation. Prognosis and decisions about intensity of care require shared discussions with families and involvement of palliative services, when appropriate. Many practices remain based on observational data or extrapolation from other populations, underlining the need for stroke-specific clinical trials. Outcomes are consistently better when patients are managed in specialized stroke or neurocritical care units with a multidisciplinary treatment approach

## 1. Introduction

Stroke is an important global health burden, as it ranks as the second main cause of death and the third leading cause of death and disability combined, globally [1]. Contemporary estimates from the World Stroke Organization’s 2025 fact sheet, obtained from the Global Burden of Disease 2021 analysis, show substantial growth in incident cases, deaths, and disability since 1990, with the largest share of mortality and DALYs occurring in low and low-middle-income countries. The global economic cost of stroke is now estimated at roughly 890 billion dollars annually, which emphasizes the challenge of its management and care [1]. Ischemic stroke accounts for the majority of cases worldwide (approximately 70–75%), whereas intracerebral hemorrhage ((ICH) 17–18%) and aneurysmal subarachnoid hemorrhage (around 8%) represent smaller but more severe and often more lethal subtypes [2,3]. Approximately 10–20% patients with acute stroke are managed in the ICU, and nearly one in five hospitalizations for acute ischemic stroke (AIS) included ICU-level care. A considerable proportion of patients with acute stroke need intensive care due to airway or ventilation needs, hemodynamic instability, malignant cerebral edema, uncontrolled intracranial pressure (ICP), or systemic complications [4]. However, ICU utilization for stroke varies widely across regions and healthcare systems. Admission thresholds depend not only on stroke severity but also on local resources, availability of neurocritical care specialists, as well as institutional protocols. Also, the clinical indications for ICU admission are inconsistently applied across centers, complicating comparisons and interpretation of outcomes. Furthermore, post-2020 shifts related to the COVID-19 pandemic have reshaped stroke ICU dynamics. Pandemic-related delays in hospital presentation and redistribution of healthcare resources increased the need for intensive monitoring and mechanical ventilation. Thrombotic complications associated with SARS-CoV-2 infection and long-COVID neurological sequelae further complicated both acute management and rehabilitation. Strokes occurring in the context of COVID-19 and post-COVID conditions have been linked with more severe presentations and a greater likelihood of hemorrhagic transformation, reported in nearly 27% of patients with long-COVID compared to about 14% in those without infection [5]. These observations reflect the lasting influence of pandemic-era changes on ICU care and recovery trajectories

Over the last decade, modern systems of care have emphasized rapid recognition and early reperfusion for AIS, alongside structured, multidisciplinary management in specialized stroke or neurocritical care units. Organized inpatient stroke unit care improves survival and functional outcomes at follow-up without lengthening hospital stay and admission to dedicated neurocritical care units and care by specialized personnel are associated with lower mortality and better functional outcomes across acute brain injuries [5,6]. Such structured approaches have shown measurable improvements in survival and independence [6,7].

Despite this, guidelines continue to evolve in parallel. For AIS, current AHA/ASA guidance emphasizes time-sensitive intravenous thrombolysis and endovascular thrombectomy when indicated, with evidence supporting tenecteplase as a practical, non-inferior alternative to alteplase in eligible patients [8]. For hemorrhagic stroke (HS), recent comprehensive guidelines from the AHA/ASA detail the acute treatment of spontaneous ICH and aneurysmal subarachnoid hemorrhage, including blood pressure targets, reversal strategies, neurosurgical considerations, and ICU-based complication prevention. The recent literature, including guideline updates, shows the ICU’s role not only in acute stabilization, but also in aligning bedside care with evolving standards of evidence-based medicine [9,10,11,12,13]. However, despite extensive guideline development, considerable heterogeneity persists between institutions and countries, particularly regarding blood pressure thresholds, timing of decompressive surgery, and use of neuromonitoring. This shows the gap between evidence-based recommendations and real-world implementation.

**Figure 1:**
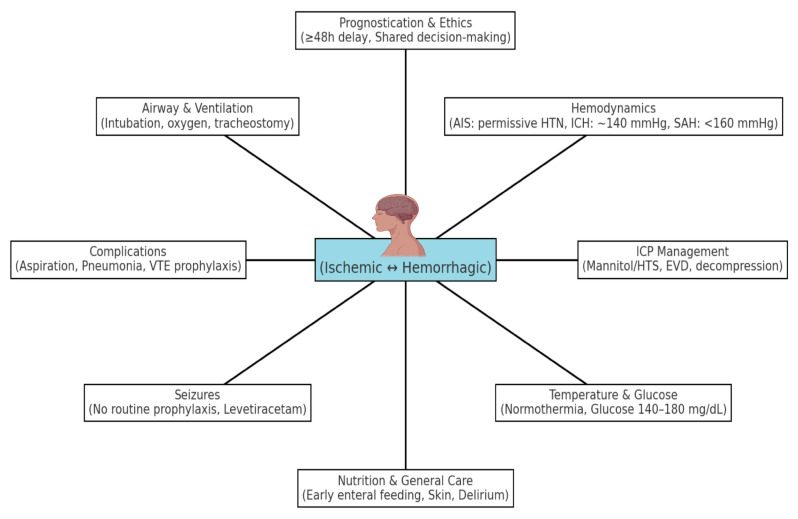
Key domains of ICU management in stroke, including airway and ventilation, hemodynamic targets (maintaining systolic BP ≤ 220/120 mmHg in untreated AIS, ≤185/110 mmHg before and ≤180/105 mmHg after thrombolysis, and ≈140 mmHg in ICH; ICP < 20–22 mmHg with CPP > 60 mmHg), intracranial pressure (ICP) control, temperature and glucose management, seizure treatment, prevention of complications, nutritional support and prognostic/ethical considerations. Abbreviations: AIS—acute ischemic stroke; ICH—intracerebral hemorrhage; SAH—subarachnoid hemorrhage; HTS—hypertonic saline; EVD—external ventricular drain; ICP—intracranial pressure; VTE—venous thromboembolism; HTN—hypertension.

In this review, we summarize current and most recent principles and best practices in ICU management of stroke patients, covering team organization, hemodynamic and physiologic targets, neurological monitoring and ICP control, seizure management, complication prevention, and considerations for prognostication and ethical decision-making (Figure 1).

## 2. Multidisciplinary ICU Care and Team Approach

Optimal stroke management in the ICU is delivered by a coordinated multidisciplinary team. Stroke patients benefit from organized stroke units and neurocritical care units staffed by specialty-trained physicians, stroke neurologists, neurosurgeons, critical care nurses, rehabilitation therapists, and other medical professionals [14]. Appropriate care in dedicated stroke units improves survival and functional recovery across stroke subtypes [6,15]. Patients receiving organized inpatient stroke unit care were more likely to survive, be independent, and stay at home one year post-stroke, regardless of age or stroke severity. Specialized neuro-ICUs have shown improved outcomes in patients with ICH, subarachnoid hemorrhage, and traumatic brain injury [14,15,16,17]. While direct proof of ICU benefits in ischemic stroke is harder to quantify, early management in comprehensive stroke centers and stroke units is well established to reduce morbidity and mortality [18].

Each discipline plays a different role: critical care physicians and neurointensivists oversee medical management and physiological optimization; stroke neurologists guide neurological monitoring and specific stroke therapies; neurosurgeons are on standby for surgical interventions (decompressive craniectomy or hematoma evacuation, for example) in malignant strokes (large hemispheric infarctions with severe cerebral edema and herniation risk), or hemorrhages. Specialized neuroscience nurses provide continuous neurologic assessments, and therapists (physical, occupational, and speech) initiate early rehabilitation and address swallowing and communication issues. Pharmacists, nutritionists, respiratory therapists, and case managers further enhance care. Regular interdisciplinary rounds and protocols ensure issues like dysphagia, mobility, and prophylaxis for complications are systematically addressed. This collaborative approach is fundamental because stroke care extends beyond acute interventions to comprehensive critical care support and early recovery efforts [19,20,21].

Nonetheless, the effectiveness of multidisciplinary models depends heavily on staffing ratios and institutional resources, which vary widely across centers. There is a lack of controlled data assessing which team compositions or intervention bundles most directly influence outcomes.

## 3. Hemodynamic and Physiological Management

### 3.1. Airway, Oxygenation, and Ventilation

Maintaining adequate oxygenation and airway protection is a first priority in stroke ICU care. Many severe stroke patients have depressed consciousness or brainstem dysfunction that can compromise airway reflexes. Endotracheal intubation is indicated for patients with loss of airway protection, significant obtundation (e.g., Glasgow Coma Scale (GCS) ≤ 8), or inability to maintain oxygenation. Prompt recognition of impending airway failure is critical to prevent aspiration, hypoxemia, and hypercapnia, because they can cause secondary neuronal injury [14,22,23]. Guidelines and recent data recommend supplemental oxygen for stroke patients if oxygen saturation falls below 94% [24,25]. Hypoxemia must be corrected swiftly, as even brief periods of low oxygen can exacerbate ischemic brain injury. Conversely, routine oxygen for non-hypoxic patients is not necessary, and hyperoxia should be avoided due to potential oxidative injury; notably, studies of hyperbaric oxygen in acute stroke showed no gain or harm [26,27,28].

Ventilatory support is tailored to patient needs. In ischemic stroke, ventilation is generally maintained at normocapnia; inadvertent hypercapnia from hypoventilation can elevate intracranial pressure (ICP), while excessive hyperventilation can reduce cerebral blood flow [29,30]. In HS or large ischemic strokes with increased ICP, short-term hyperventilation may be used as a temporizing measure to reduce ICP through cerebral vasoconstriction, but this strategy is reserved for acute neurological deterioration given the associated risk of cerebral ischemia [31]. Patients who develop acute respiratory distress or neurogenic pulmonary edema, a recognized complication after subarachnoid hemorrhage, may require higher levels of ventilatory support and positive end-expiratory pressure (PEEP), with careful titration to balance oxygenation against potential effects on intracranial dynamics and hemodynamics [32,33]. For patients requiring prolonged mechanical ventilation, tracheostomy is frequently considered. The optimal timing remains uncertain, as the SETPOINT-2 randomized controlled trial compared early (≤5 days) and standard (≥10 days) tracheostomy in patients with severe stroke and found no meaningful difference in survival without severe disability at 6 months [34]. Nevertheless, early tracheostomy may still provide benefits such as improved patient comfort, reduced sedation needs, and facilitation of ventilator weaning in appropriately selected cases [35]. Still, the evidence remains conflicting, and decisions on timing are best individualized, because randomized trials have not yet confirmed the functional advantages suggested by observational data.

Careful respiratory monitoring, avoidance of aspiration, and timely intubation are central to stroke ICU care, as hypoxic or aspirational injury can dramatically worsen neurological outcomes [36]. However, trials like SETPOINT-2 were conducted in specialized ICUs and may not reflect outcomes in lower-resource environments. The optimal timing and patient selection for tracheostomy are still uncertain, and it needs to be clarified which subgroups can truly benefit.

### 3.2. Blood Pressure

Blood pressure (BP) control in stroke must be individualized to stroke type and phase of care, aiming to support cerebral perfusion in ischemic tissue while avoiding extremes that could worsen edema or bleeding [37].

#### 3.2.1. Ischemic Stroke (AIS)

Patients often present with elevated BP due to endogenous autoregulatory responses to cerebral ischemia [38]. Permissive hypertension is generally allowed in the early phase for patients who have not received reperfusion therapy to optimize collateral blood flow to the ischemic penumbra. Current American Heart Association (AHA)/American Stroke Association guidelines suggest not treating BP unless it exceeds 220/120 mmHg in the first 24–48 h for AIS patients who are not thrombolysis or thrombectomy candidates [17,38]. Lowering BP too aggressively in this context may reduce cerebral perfusion and worsen the stroke. However, if the patient is a candidate for thrombolytic therapy (IV alteplase), stricter BP control is required: BP must be reduced to ≤185/110 mmHg before tPA administration and kept at ≤180/105 mmHg for at least the first 24 h after thrombolysis to reduce the risk of intracerebral hemorrhagic transformation. Similarly, during and after endovascular thrombectomy for large-vessel occlusion, guidelines recommend maintaining BP ≤180/105 mmHg [39,40,41]. After successful reperfusion, moderate systolic BP targets around 120–160 mmHg may help reduce reperfusion injury. Trials have not shown the benefit of lowering below 120 mmHg, and ENCHANTED2/MT even reported worse outcomes with such intensive reduction [42]. Current practice favors individualized targets while avoiding both severe hypertension and excessive hypotension. In all AIS patients, hypotension and hypovolemia should be avoided, as low systemic pressure can critically reduce cerebral perfusion in penumbral regions. Volume depletion should be corrected (preferably with isotonic fluids) and precipitating causes of hypotension identified (cardiac arrhythmias, myocardial infarction, or sepsis) with the help of point-of-care ultrasound and other tools [43,44]. Hypotonic fluids (dextrose-containing or half-normal saline) are avoided in neurocritical care because they can exacerbate cerebral edema. In select AIS patients with neurologic worsening due to presumed low-flow states (for instance, hemodynamic strokes or vasospasm), pharmacologically induced hypertension has been attempted to augment cerebral perfusion, though its efficacy remains unproven [45].

#### 3.2.2. Hemorrhagic Stroke (HS)

In ICH, elevated BP is very common and linked to hematoma expansion. Randomized trials provide mixed results: INTERACT-2 showed that reducing systolic BP to <140 mmHg soon after onset is safe and may modestly improve outcomes, while ATACH-II found no benefit from lowering below 130 mmHg compared with <180 mmHg [46]. These conflicting findings suggest that intensive reduction below 140 mmHg may not provide additional benefit and could even increase the risk of hypoperfusion. A systolic target around 140 mmHg is therefore generally regarded as a reasonable and safe approach, with clinical judgment remaining essential, particularly in patients with borderline perfusion. Excessive lowering (<120 mmHg) should be avoided, as it may compromise perihematomal perfusion [46]. In practice, many clinicians adopt a middle-ground approach, but the lack of definitive evidence leaves this area open to debate and further study.

For aneurysmal SAH, high BP raises the risk of aneurysm rebleeding before the aneurysm is secured. Guidelines have long advised prompt control of blood pressure in SAH, typically aiming for a systolic BP of <160 mmHg until the aneurysm is obliterated, to strike a balance between preventing rebleeding and maintaining cerebral perfusion [47]. Intravenous titratable agents (like nicardipine or labetalol) are used to keep SBP in a safe range (often 140–150 mmHg) prior to aneurysm clipping or coiling [48]. Once an aneurysm is secured, attention shifts toward preventing delayed cerebral ischemia from vasospasm. At that stage, blood pressure is often supported at normal to high-normal levels (permissive hypertension) to optimize cerebral perfusion [47,48].

BP management in stroke requires dynamic adjustment: in ischemic stroke, avoid extremes and allow higher pressures initially (unless treating with reperfusion therapies), whereas in HS, early aggressive BP lowering (to ~140 mmHg in ICH or <160 mmHg in SAH) is indicated to limit bleeding, with vigilance to avoid hypotension in all cases [18,46].

### 3.3. Temperature and Glycemic Control

Fever is unfavorable in acute brain injury, as even moderate hyperthermia can worsen ischemic damage and increase intracranial pressure. Post-stroke fever (core temperature > 37.5 °C) is associated with greater mortality and worse functional outcomes. The goal is to maintain normothermia, typically 36.0–37.5 °C, with aggressive treatment of any temperature elevation. This includes antipyretics and evaluation for infection, while refractory central fever may require external or endovascular cooling devices. Therapeutic hypothermia has not demonstrated clinical benefit in stroke; early trials were inconclusive, and the DEPTH-SOS trial was stopped for potential harm in the hypothermia group [49]. Accordingly, current guidelines emphasize fever prevention and maintenance of normothermia rather than induction of hypothermia [50,51]. The failure of therapeutic hypothermia in clinical trials despite promising preclinical evidence reflects a broader challenge in translating neuroprotective strategies from laboratory to bedside.

Hyperglycemia is common after AIS, even in patients without diabetes, and is associated with worse neurological outcomes and increased mortality. Elevated glucose may exacerbate injury through lactic acidosis and oxidative stress, whereas hypoglycemia is also harmful and must be avoided. The SHINE randomized trial found no improvement in outcomes with intensive insulin therapy (80–130 mg/dL) compared with a more moderate target (80–180 mg/dL), but significantly more hypoglycemic events [52]. Similar results were seen a while ago in the NICE-SUGAR trial in critically ill patients, where intensive targets increased mortality [53]. Current practice, therefore, aims for a blood glucose range of 140–180 mg/dL (7.8–10 mmol/L), treating persistent hyperglycemia with insulin while promptly correcting any hypoglycemia. The overarching goal is to avoid extremes of blood sugar, supporting metabolic stability in the injured brain [54]. Yet, evidence for strict glucose control and therapeutic hypothermia has been largely inconclusive or even harmful, which reflects a gap between physiologic rationale and clinical outcomes.

### 3.4. Other Physiological Considerations

Ensuring euvolemia and normonatremia is particularly important in neurocritical care. Dehydration can worsen cerebral ischemia, while fluid overload may aggravate cerebral edema. Generally, isotonic crystalloids (normal saline) are used for maintenance and volume repletion in stroke patients. Hypotonic fluids are contraindicated in the acute phase because they can cause hyponatremia with increased brain swelling. After subarachnoid hemorrhage (SAH), patients are prone to salt-wasting and hypovolemia, which can contribute to cerebral vasospasm; thus, vigilant monitoring of fluid balance and serum electrolytes (especially sodium) is required, with replacement of deficits to maintain normal volume status. Avoidance of hypotonic hyponatremia (from syndrome of inappropriate antidiuresis or cerebral salt-wasting) is critical, as hyponatremia can exacerbate cerebral edema and precipitate seizures. In SAH, earlier practices of inducing hypervolemia to prevent vasospasm (“triple-H therapy”) have been abandoned in favor of simply maintaining normal volume and blood pressure, since prophylactic hypervolemia showed no clear benefit [55,56,57,58]. When vasospasm occurs despite preventive measures, management focuses on maintaining adequate cerebral perfusion and preventing secondary ischemia. Induced hypertension remains the mainstay of therapy when tolerated hemodynamically, while euvolemia is preserved. Oral or intravenous nimodipine is routinely administered to reduce the risk and severity of delayed cerebral ischemia and improve outcomes after aneurysmal SAH. In refractory cases, endovascular options such as intra-arterial vasodilator infusion (e.g., verapamil and nicardipine) or balloon angioplasty may be considered in specialized centers [59,60].

Stroke patients, like other critical patients, should start enteral nutrition early (ideally during the initial 48 h) to prevent catabolism and support healing. Early feeding (via nasogastric or nasoenteric tube if the patient is unable to swallow safely) is considered safe and may modestly improve survival in dysphagic stroke patients, supporting the importance of timely nutritional support [61]. Conversely, prolonged fasting can worsen weakness and immune dysfunction. In patients with severe dysphagia, a post-pyloric feeding tube (nasoduodenal) may reduce aspiration risk by bypassing the stomach. Nutritional therapy in the ICU should also account for energy balance and glucose control. Caloric intake is generally guided by indirect calorimetry or weight-based estimates, with a target of 25–30 kcal/kg/day. Overfeeding and rapid glucose fluctuations should be avoided, as both can worsen metabolic stress and impair recovery. Enteral formulas enriched with protein and antioxidants are preferred, while parenteral nutrition is reserved for cases where the enteral route is not feasible or contraindicated [62].

Stress ulcer prophylaxis with H2-blockers or proton pump inhibitors is often given to stroke ICU patients who are intubated or have risk factors for gastrointestinal bleeding, although the overall benefit must be weighed against any risk of pneumonia from higher gastric pH (the evidence is mixed, so practice varies by institution). Care is also taken to avoid excessive sedation and to promote regular sleep–wake cycles to reduce delirium, as delirium can complicate the neurologic examination and prolong ICU stay [63,64,65].

### 3.5. Management of Comorbid Conditions

Comorbidities are highly prevalent among stroke ICU patients and significantly influence outcomes. Hypertension, diabetes mellitus, ischemic heart disease, and atrial fibrillation often coexist and require careful adjustment of ongoing therapies. Antihypertensive treatment should maintain adequate cerebral perfusion while preventing excessive blood pressure variability. Glucose control must balance the risks of hyperglycemia-related complications with the dangers of hypoglycemia, particularly in patients receiving enteral nutrition or insulin infusions. Cardiac monitoring allows early detection of arrhythmias or myocardial ischemia, and management should be individualized in collaboration with cardiology and endocrinology teams. Integrating comorbidity management into routine ICU protocols supports hemodynamic stability, reduces systemic complications, and facilitates safer transition to step-down or ward care [66,67].

## 4. Neurological Monitoring and Intracranial Pressure Management

Close neurological monitoring is essential in the stroke ICU. Standardized assessments (National Institutes of Health Stroke Scale (NIHSS), GCS, and pupillary checks) are performed frequently to detect early deterioration, prompting rapid imaging and intervention. Neuroimaging is integral to ICU stroke care, allowing early detection of hematoma expansion, edema, or hydrocephalus. Repeat CT or MRI is warranted with neurological decline or after interventions to assess treatment response, while bedside imaging can minimize transport risks in unstable patients [68,69]. In selected cases, invasive neuromonitoring such as external ventricular drain (EVD) placement provides both ICP measurement and therapeutic CSF drainage, particularly in HSs with hydrocephalus [70,71]. Invasive monitoring of ICP can be performed using intraventricular catheters or intraparenchymal probes, depending on local expertise and indication. Intraventricular monitoring remains the gold standard as it allows both pressure measurement and cerebrospinal fluid drainage, while intraparenchymal devices offer lower infection risk but do not permit drainage. Target ICP values are generally maintained below 20–22 mmHg, with cerebral perfusion pressure (CPP) above 60 mmHg to ensure adequate cerebral blood flow. These thresholds are extrapolated from traumatic brain injury data but are commonly applied in stroke-related intracranial hypertension to guide osmotherapy and surgical decision-making [72,73].

Continuous EEG monitoring is also useful in observing subclinical seizures in patients with altered consciousness [74]. The lack of high-quality data linking continuous EEG detection of subclinical seizures to improved outcomes is a major limitation. Most supporting evidence is observational, and practice patterns vary widely. This uncertainty complicates guideline implementation, since resource-intensive monitoring may not translate into measurable benefits in all settings.

ICP management follows a tiered strategy. General measures include head-of-bed elevation, midline positioning, sedation and analgesia, and avoidance of venous outflow obstruction or agitation. Osmotic therapy with mannitol/hypertonic saline is first-line for acute ICP elevations, while prophylactic use is not recommended. Hyperventilation may provide transient relief but is reserved as a bridge to definitive treatment. Corticosteroids are ineffective and potentially harmful in stroke-related edema and are not recommended [75,76].

In ischemic stroke, malignant cerebral edema after large MCA infarction carries high mortality with medical therapy alone. Early decompressive hemicraniectomy within 48 h considerably lowers mortality and increases chances of functional survival, especially in patients ≤60 years. In older patients, surgery improves survival but often with severe disability, highlighting the importance of individualized decision-making. Posterior fossa decompression or EVD may be lifesaving in large cerebellar infarctions with brainstem compression [77,78].

In ICH, ICP may rise due to hematoma mass effect and edema. Ventriculostomy is indicated for hydrocephalus, improving survival. Large cerebellar hemorrhages (>3 cm) or those causing brainstem compression and/or hydrocephalus require urgent surgical evacuation. In supratentorial ICH, benefits of surgery are less clear; minimally invasive approaches, stereotactic aspiration or thrombolysis (MISTIE III), are still under study. Intraventricular hemorrhage with obstructive hydrocephalus is managed with EVD, sometimes with intraventricular fibrinolysis, though outcome benefit remains uncertain [75,79].

Across all stroke types, refractory ICP may require deep sedation, neuromuscular blockade, or barbiturate coma in selected cases. Timely recognition of neurologic decline and a structured ICP management algorithm (combining supportive measures, targeted osmotherapy and timely neurosurgical intervention) are key to preventing herniation and improving survival [80,81,82]. However, much of the evidence for ICP-directed therapy in stroke is extrapolated from traumatic brain injury cohorts. Large randomized trials specifically addressing invasive ICP monitoring in ischemic or HS are lacking, so the true effect on long-term functional outcomes remains uncertain. This raises questions about whether current practices are evidence-driven or largely tradition-based. The absence of robust randomized data also makes it difficult to determine cost-effectiveness and patient selection criteria for invasive ICP monitoring in stroke, in contrast to the more established evidence base in traumatic brain injury.

## 5. Seizure Prevention and Management

Stroke, both ischemic and hemorrhagic, can provoke seizures due to acute cortical injury, and seizures can contribute to secondary brain damage by raising metabolic demand and ICP. Management involves treating any occurring seizures promptly and the judicious use of prophylactic antiseizure medications. The approach differs slightly between ischemic and HS [82,83,84]. Incidence varies by stroke type: early seizures occur in 2.7–30% of ICH, particularly with lobar or cortical location, large hematoma volume, and younger age. In aneurysmal subarachnoid hemorrhage, 5–10% present with seizures, with additional risk during delayed cerebral ischemia. Ischemic stroke carries a lower risk (3–5%), mainly in large cortical or embolic infarcts. Late post-stroke epilepsy is a distinct chronic complication outside the ICU setting [85,86,87,88,89].

When seizures do occur in the stroke unit or ICU, prompt therapy is essential. Prolonged convulsions are treated first with intravenous benzodiazepines, typically lorazepam, followed by initiation of an antiseizure drug. Levetiracetam has become the agent of choice in this context because of its favorable tolerability and lack of drug interactions, gradually replacing phenytoin in most neurocritical care settings. Status epilepticus, though rare, requires escalation to continuous EEG monitoring, high-dose antiseizure therapy, and, in refractory cases, anesthetic infusions in accordance with established ICU protocols [89,90,91,92].

A central management question concerns the role of prophylactic antiseizure medication in stroke patients who have not seized. For both ICH and AIS, the consensus of clinical evidence and contemporary guidelines is to avoid prophylactic use, given the absence of demonstrated benefit and the potential for harm from unnecessary sedation and cognitive adverse effects. The 2022 AHA/ASA guideline for ICH management explicitly advises against routine prophylaxis (Class III, no benefit). Subarachnoid hemorrhage represents a partial exception: although early practice favored prophylactic phenytoin for up to one week, subsequent studies revealed worse outcomes and no seizure-preventive effect. As a result, current recommendations discourage routine long-term prophylaxis, allowing only short-term, selective administration of levetiracetam in high-risk situations, such as perioperative seizures or concomitant intracerebral hematoma [75,83,93]. The absence of large RCTs, specifically evaluating prophylaxis in stroke, means that current recommendations rest on indirect evidence and observational data. Whether specific high-risk subgroups, such as lobar ICH with cortical involvement, could benefit from tailored prophylactic strategies is still unresolved.

Because many critically ill stroke patients are sedated or encephalopathic, seizures may manifest only as subtle mental status changes. Continuous EEG monitoring is therefore increasingly applied in patients with unexplained neurological depression, in order to detect non-convulsive seizures and electrographic status epilepticus [94,95]. While direct evidence that treating subclinical seizures improves outcomes is difficult to establish, experts state that ongoing epileptiform activity is detrimental and warrants intervention [94,95]. However, the role of prophylactic antiseizure drugs in stroke remains unresolved. Regional practice varies considerably, and well-designed multicenter randomized trials are required to establish clear guidance for high-risk populations.

## 6. Prevention and Management of ICU Complications

Stroke patients in the ICU are vulnerable to various medical complications that can affect recovery. Comprehensive critical care includes strategies to prevent, detect, and treat these complications. Key issues are protecting against aspiration and pneumonia, preventing venous thromboembolism, managing infections, and maintaining skin, joints, and overall medical stability [37,96,97].

Dysphagia is common after stroke, particularly with brainstem or cranial nerve involvement, and predisposes to aspiration and pneumonia, which is also one of the leading causes of morbidity and/or mortality in these patients [97]. Stroke-associated pneumonia occurs in roughly 14% of patients overall, with much higher rates in severe and ICU cases. Its development is linked to longer hospital stays and several-fold higher mortality [98,99,100]. Early dysphagia screening before any oral intake is a universal recommendation. Bedside water swallow tests or standardized protocols, often conducted by nurses or speech therapists, guide safe feeding decisions. Patients who fail screening should receive tube feeding until formal evaluation; use of standardized screening protocols has been associated with lower pneumonia rates [101,102].

For those with impaired swallowing or consciousness, strict aspiration precautions are applied: maintaining the head of the bed elevated, suctioning oral secretions, withholding unsafe oral intake, and ensuring rigorous oral hygiene to reduce bacterial colonization. In ventilated patients, ventilator care bundles including head-of-bed elevation, sedation breaks, and subglottic suction are equally important. Nutritional support should begin within 48 h, typically via nasogastric tube, though post-pyloric feeding may be considered in high aspiration risk. Persistent dysphagia often necessitates PEG placement after two weeks. Proper nutrition prevents catabolism, supports wound healing, and strengthens immune function [103,104,105].

If pneumonia develops, prompt broad-spectrum antibiotics, airway clearance measures, and ventilatory support are the most important treatment plan. Trials of routine prophylactic antibiotics (for example, Preventive Antibiotics in Stroke Study (PASS)) [106] did not reduce pneumonia incidence or improve outcomes and are therefore not recommended. Instead, emphasis remains on non-pharmacologic strategies such as dysphagia management, oral care, early mobilization, and vigilant pulmonary hygiene [107,108,109]. To this day, implementing systematic dysphagia screening and oral care protocols is uneven in clinical practice, particularly outside high-income centers. This variation in adherence to simple preventive measures underscores a global implementation gap, many evidence-based practices fail to reach bedside due to resource or training constraints.

## 7. Venous Thromboembolism Prophylaxis

Immobility after stroke, especially in hemiplegic patients, greatly increases the risk of deep vein thrombosis (DVT) and pulmonary embolism (PE). Without prophylaxis, the incidence of DVT in stroke patients can be significant (in older studies, asymptomatic DVT was found in up to 50% of hemiparetic legs by 2 weeks post-stroke if no prophylaxis was given). Preventing venous thromboembolism (VTE) is thus a priority in the ICU [110,111,112]. All immobilized stroke patients should receive mechanical DVT prophylaxis on admission. Intermittent pneumatic compression (IPC) devices significantly reduce DVT and improve survival. In contrast, graduated compression stockings showed no benefit in CLOTS 1 and 2 and are not recommended [113,114].

Pharmacologic prophylaxis with low-dose heparin or low molecular weight heparin (LMWH) is highly effective but must be timed carefully. In ischemic stroke, prophylaxis is typically started within 24 h if no contraindications exist and at least 24 h after thrombolysis. In ICH, low-dose heparin or LMWH may be considered at 24–48 h once follow-up imaging confirms hematoma stability. Thus, chemical prophylaxis is favored within 1–3 days after ICH when stable, while relying on IPC initially [115,116]. If DVT or PE occurs before anticoagulation is safe, temporary IVC filter placement may be used as a bridge. Full-dose anticoagulation is usually initiated after a few days in ischemic stroke or after 1–2 weeks in ICH, depending on bleeding stability [117,118]. Early IPC plus timely low-dose heparin remains the standard VTE prevention strategy in stroke, reducing the risk of potentially fatal PE while balancing hemorrhagic risk.

Antithrombotic management in the ICU setting demands a careful and individualized strategy balancing the prevention of ischemic and embolic events against the risk of hemorrhagic complications. In AIS, antiplatelet therapy is typically initiated once hemorrhagic transformation has been excluded (e.g., via 24 h imaging), aspirin (75–325 mg daily) is the cornerstone, while short-term dual antiplatelet therapy (e.g., aspirin + clopidogrel for up to 21 days) may be considered in selected patients with minor non-cardioembolic strokes or high-risk TIA [119]. In patients with atrial fibrillation and acute cardioembolic stroke, the timing of oral anticoagulation restart is guided by infarct size, hemorrhagic risk, and imaging stability. Some expert models (e.g., “1-3-6-12-day” rule) suggest resuming anticoagulants at day 1 for TIA, day 3 for small infarct, day 6 for moderate, and day 12 for severe infarcts, although high-quality randomized data are lacking [120]. After an intracerebral hemorrhage, antithrombotic therapy is often withheld during the acute phase; re-initiation (either antiplatelet or anticoagulant) depends on hematoma stability, imaging findings (absence of growth or contrast extravasation), and thromboembolic risk profile (mechanical valve, atrial fibrillation, or large-vessel disease). Multidisciplinary discussion with neurology, hematology, and cardiology is recommended in this setting. Lastly, mechanical VTE prophylaxis combined with low-dose heparin or LMWH remains a standard of care in immobilized stroke ICU patients, but must be coordinated with the timing of reperfusion therapies and intracranial bleeding risk [121,122].

## 8. Other Medical Complications and General Care

Beyond neurological care, stroke patients in the ICU are highly vulnerable to medical complications that can complicate recovery. Infections are frequent, particularly UTIs and sepsis, often related to urinary catheters. Indwelling catheters should be avoided or removed early, with intermittent catheterization preferred. Stroke-induced immune depression further increases infection risk, warranting vigilant monitoring and early antibiotic treatment when indicated [106,123]. Pressure ulcers are prevented through regular repositioning, skin inspection, and pressure-relieving surfaces. Joint contractures and muscle atrophy are limited by range-of-motion exercises, splints, and early mobilization. Rehabilitation should begin within 24–48 h, with short, frequent sessions; very early and aggressive mobilization (<24 h) has shown no benefit and may be harmful [124]. Delirium is addressed through reorientation, day–night regulation, early mobilization, and careful use of sedatives. Sympathetic storming, though rare, can occur after severe strokes and is managed supportively [125].

Best practice in ICU stroke care emphasizes infection control, immobility prevention, skin protection, early rehabilitation, and comprehensive supportive care. These measures are part of modern stroke center protocols and are associated with improved outcomes and reduced mortality [123,124,125,126].

A concise overview of the above-discussed best practices in ICU stroke care is presented below in Table 1.

## 9. Prognostication, Palliative Care, and Ethical Considerations

Severe strokes often carry a high risk of death or major disability, forcing difficult ICU decisions regarding the intensity of care and palliative care preferences. Accurate prognostication is especially uncertain in the first 24–48 h, and early pessimism can lead to self-fulfilling withdrawal of care. On the other hand, unrealistic optimism may prolong invasive treatments misaligned with patient values. Guidelines recommend continuing aggressive care for at least the first 1–2 days before considering new Do Not Resuscitate (DNR) orders or withdrawal of support [126,127]. Prognostic tools such as the ICH Score or NIHSS provide population-level estimates but cannot predict outcomes for individuals. But, these scores were developed decades ago and may not reflect modern treatment paradigms or the impact of neurocritical care interventions, warranting revalidation in contemporary cohorts. Stroke trajectories are variable and some patients exceed grim early expectations. Clinicians must acknowledge uncertainty, avoid deterministic predictions, and revisit prognosis as the course evolves [128,129].

Shared decision-making with families is also important, since many patients cannot speak for themselves. Clear discussions about goals of care, code status, and interventions (feeding tubes and tracheostomy) should be framed by the patient’s prior wishes or values. Palliative care teams improve communication, provide symptom control, and support families; their early involvement is highly encouraged [130]. Withdrawal of life-sustaining therapy (WLST) is very common in devastating strokes, but should never be rushed. If chosen, it is performed collaboratively with surrogates, ensuring comfort measures and psychosocial support. DNR orders must be correctly understood as applying only to resuscitation, not as a signal to limit other indicated treatments [131].

Ethical principles of autonomy, beneficence, and non-maleficence guide decision-making. Conflicts between families and providers may require ethics consultation. Balancing hope for recovery with realism about likely outcomes, while aligning treatment to patient values, is central to high-quality stroke ICU care [132].

## 10. Conclusions

Management of stroke in the ICU demands a coordinated, evidence-based approach that balances acute neurological stabilization with prevention of systemic complications. Key priorities include airway protection, individualized blood pressure control, strict maintenance of normothermia and normoglycemia, seizure surveillance and ICP management, with timely surgical interventions when indicated. Preventing aspiration, pneumonia, venous thromboembolism, and other ICU-related complications is equally critical, supported by early nutrition and rehabilitation. At the same time, much of current practice still relies on extrapolation from other critical care populations or on observational data rather than stroke-specific randomized evidence. This is evident in areas such as seizure prophylaxis, continuous EEG monitoring, and optimal blood pressure targets in ICH. These gaps limit the certainty with which guidelines can be applied and create heterogeneity in practice worldwide. Prognostication remains particularly challenging in the acute phase; decisions about the intensity of care should be guided not only by clinical tools but also by patient values, shared discussions with families, and timely integration of palliative care. Ultimately, outcomes are consistently better when patients are managed in specialized stroke or neurocritical care units with multidisciplinary expertise, but sustained progress will require targeted trials to address unresolved questions and refine best practices in the ICU setting. Beyond the acute phase, comprehensive post-discharge care is essential for recovery and secondary prevention. Early initiation of rehabilitation, optimization of vascular risk factors, and structured follow-up after ICU discharge support functional improvement and reduce recurrence. Coordinated transition planning between critical care, neurology, and rehabilitation teams ensures continuity of care and long-term stability.

## Figures and Tables

**Table 1:** Overview of recommended, avoided, and cautionary practices in stroke ICU management.

Domain	Dos (Recommended)	Don’ts (Avoid)	Cautions
Blood Pressure Management	Maintain SBP ≤ 220/120 mmHg in AIS patients not receiving reperfusion therapy, or ≤185/110 mmHg before and ≤180/105 mmHg after thrombolysis; maintain SBP ≈ 140 mmHg in ICH patients; use titratable IV agents (e.g., nicardipine, labetalol)	Rapid BP drops <120 mmHg	Individualize targets based on perfusion status and stroke subtype
Glycemic Control	Keep glucose 140–180 mg/dL; treat persistent hyperglycemia with insulin	Intensive insulin protocols (80–110 mg/dL)	Prevent hypoglycemia, especially in sedated or enterally fed patients
Temperature Management	Maintain normothermia; treat fever >37.5 °C with antipyretics or cooling	Unnecessary therapeutic hypothermia	Monitor for shivering and metabolic stress
Seizure Management	Use EEG for unexplained decline in consciousness or neurological status; treat only confirmed seizures or status epilepticus	Routine prophylaxis in ischemic stroke	Consider prophylaxis short-term in ICH with cortical involvement
Antithrombotic Therapy	Start antiplatelet (aspirin) 24–48 h after excluding bleeding; time anticoagulant restart based on imaging and infarct size	Early full-dose anticoagulation post-ICH	Multidisciplinary assessment of bleeding vs. thrombotic risk
Intracranial Pressure and Fluids	Maintain ICP < 20–22 mmHg and CPP > 60 mmHg; use osmotherapy if elevated	Hypotonic fluids	Ensure CPP > 60 mmHg; monitor sodium closely
Nutrition and General Care	Start enteral feeding within 48 h; use protein-rich formulas	Prolonged fasting or overfeeding	Balance caloric intake with glucose control

## Data Availability

No new data were created or analyzed. Data sharing is not applicable.
